# Identification of Potential Therapeutic Agents for Type I Interferonopathy Using iPSC-Based Disease Modeling

**DOI:** 10.1007/s10875-025-01933-8

**Published:** 2025-09-30

**Authors:** Bunki Natsumoto, Hirofumi Shoda, Motonori Tsuji, Makoto Otsu, Hideki Taniguchi, Kazuhiko Yamamoto, Keishi Fujio

**Affiliations:** 1 Department of Allergy and Rheumatology, Graduate School of Medicine, The University of Tokyo, Tokyo, Japan; 2 Department of Rheumatology, Tokyo Medical University Hospital, Tokyo, Japan; 3 Institute of Molecular Function, Saitama, Japan; 4 Division of Hematology, Department of Medical Laboratory Sciences, Kitasato University School of Allied health Sciences, Kanagawa, Japan; 5 Division of Stem Cell Processing/Stem Cell Bank, Center for Stem Cell Biology and Regenerative Medicine, Institute of Medical Science, University of Tokyo, Tokyo, Japan; 6 Laboratory for Autoimmune Diseases, RIKEN Center for Integrative Medical Sciences, Yokohama, Japan

**Keywords:** Interferonopathy, Aicardi-goutières syndrome (AGS), Induced pluripotent stem cells (iPSCs), Interferon induced with helicase c domain 1 (*IFIH1*), *In Silico* prediction, Oligoadenylate synthetase (OAS) family

## Abstract

**Purpose:**

Type I interferonopathy encompasses disorders marked by systemic inflammation and neurological involvement, arising from genetic mutations that result in the upregulation of type I IFN signaling through various mechanisms. Currently, therapeutic options are limited, and no standard therapy exists. This study aims to develop a strategy for identifying new therapeutic targets for type I interferonopathy using induced pluripotent stem cells (iPSCs).

**Methods:**

The *IFIH1* R779H variant was introduced into iPSCs through genome editing. RNA sequencing of iPSC-derived dendritic cells (DCs) was performed, and differentially expressed genes (DEGs) were identified. IFN-α secretion, reactive oxygen species (ROS), and mitochondrial oxygen consumption rate (OCR) were analyzed in iPSC-derived DCs. An in silico prediction of compounds binding to the OAS-like domain was conducted. Candidate compounds were evaluated for their ability to inhibit IFN secretion from *IFIH1* R779H-mutated iPSC-derived DCs.

**Results:**

Transcriptome analysis indicated upregulation of the IFN-related and metabolic pathways. *IFIH1* R779H-mutated iPSC-derived DCs exhibited increased OCR and ROS generation, and blocking mitochondrial metabolism significantly reduced excessive IFN-α secretion. Among the DEGs, *PML* was upregulated, and targeting this gene with arsenic trioxide (ATO), a PML antagonist, suppressed IFN-α secretion from *IFIH1* R779H-mutated iPSC-derived DCs. Additionally, bisantrene, phthalylsulfathiazole and ganaplacide were predicted to bind to the RNA binding groove of OAS-like domain of human OASL in silico, effectively inhibiting IFN-α secretion from *IFIH1* R779H-mutated DCs.

**Conclusion:**

Our iPSC-based disease modeling and drug investigation approach provides a robust platform for validating the efficacy and toxicity of candidate therapeutic agents for rare and intractable human diseases such as type I interferonopathy.

**Graphical Abstract:**

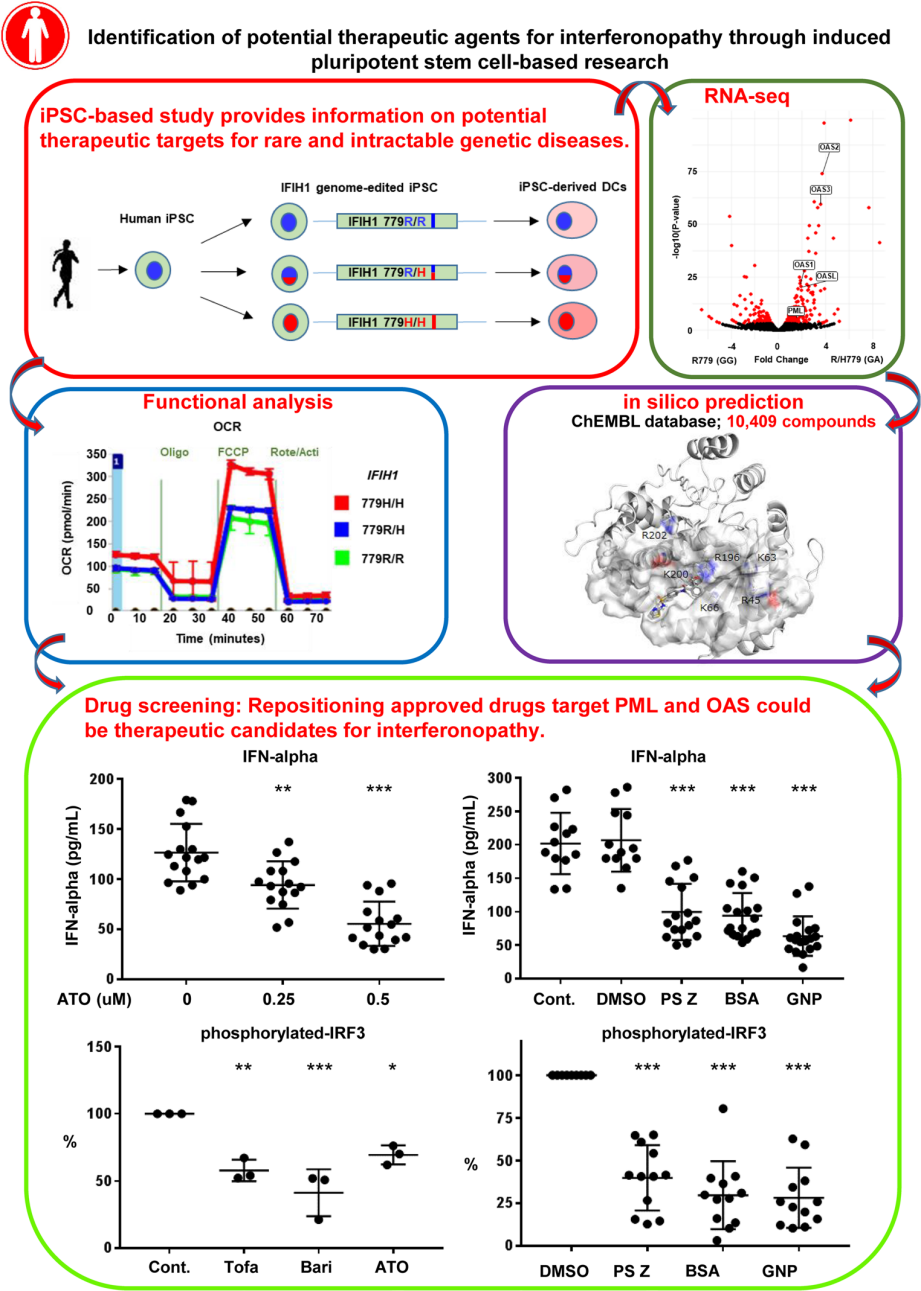

**Supplementary Information:**

The online version contains supplementary material available at 10.1007/s10875-025-01933-8.

## Introduction

Type I interferonopathy is a type of autoinflammatory disease characterized by excessive production of interferons (IFNs) due to genetic mutations [1, 2]. For example, patients with Aicardi-Goutières Syndrome (AGS), a representative disease of type I interferonopathy, experience infantile-onset mental and neurological disturbances, accompanied by systemic inflammation. Numerous genetic mutations have been identified as causes of type I interferonopathy [1, 2]. These genes are typically associated with nucleic acid metabolism, nucleic acid sensing, the immunoproteasome, and signal transduction. AGS is a well-known example of type I interferonopathy [3]. AGS is an inherited encephalopathy that appears in newborns and infants, presenting with mental and neurological symptoms. Additionally, patients often display puffy swelling and chilblains in their fingers due to small vessel inflammation. Some individuals with AGS also develop autoimmune disorders, such as systemic lupus erythematosus (SLE), which is referred to as monogenic lupus [4]. Notably, since the IFN response plays a key pathogenic role in SLE, it is broadly considered a form of type I interferonopathy [5, 6]. Currently, there are no curative treatments available for AGS, and the prognosis depends on the severity of the disease. In particular, CNS manifestations are the major cause of mortality and morbidity in AGS, and these symptoms are largely irreversible once established. As a result, therapeutic development for AGS often focuses on alleviating specific symptoms and managing comorbidities, rather than fully reversing the disease. This highlights the need for early intervention based on a deep understanding of disease mechanisms.

Therefore, in this study, we sought to model AGS using iPSCs carrying the *IFIH1* R779H mutation to investigate the pathophysiology of the disease and identify potential therapeutic agents that could modulate excessive IFN signaling before irreversible damage occurs. Mutations in various genes, including Interferon Induced with Helicase C Domain 1 (*IFIH1*), have been linked to AGS [3, 7]. These mutations disrupt nucleic acid metabolism and immune responses, leading to excessive IFN production in both systemic and central nervous systems. Baricitinib, a JAK1/2 inhibitor, is currently being evaluated in clinical trials for the treatment of AGS [8]. Since JAK1/2 are adaptor kinases downstream of IFN receptors, inhibiting IFN signal transduction is a promising curative strategy for AGS [8]. However, further research is needed to develop curative therapies for interferonopathies.

Recently, induced pluripotent stem cells (iPSCs) have emerged as a valuable tool for drug screening and development. Patient-derived iPSCs with genetic mutations can be used to investigate both disease mechanisms and potential therapies [9]. Additionally, genome editing enables the creation of iPSCs with specific causal mutations. There have been several successful cases of drug screening using iPSCs in the context of genetic diseases [10, 11]. iPSCs offer distinct advantages for disease modeling and drug screening. Unlike primary cells, iPSCs can be expanded repeatedly in large quantities, and human cell types that are difficult to obtain, such as neural cells, can be generated through in vitro differentiation [9]. This makes iPSCs a promising platform for disease modeling and drug screening in genetic immune diseases. In previous work, we established iPSCs carrying the *IFIH1* R779H mutation using genome editing [12]. *IFIH1* encodes melanoma differentiation-associated protein 5 (MDA5), a cytosolic receptor for double-stranded (ds) RNA that promotes the expression of IFN-stimulated genes (ISGs) [7]. The *IFIH1* R779H mutation can cause AGS or child-onset SLE [7]. A previous report demonstrated that *IFIH1* R779H mutation confers a gain-of-function effect, with the mutant MDA5 binding more avidly to RNA and leading to increased interferon signaling both with and without ligand stimulation [7]. Indeed, we previously reported that dendritic cells (DCs) derived from iPSCs with the *IFIH1* R779H mutation spontaneously secreted elevated levels of type I IFNs and exhibited heightened responses to dsRNA stimulation [12]. This iPSC-derived DC exhibited plasmacytoid DC-like phenotypes, such as surface marker CD123 and CD303 positivity, as well as an increased IFN-secreting potential in response to nucleolar ligands [12]. Since plasmacytoid DCs are considered as a major source of type Ⅰ IFNs in autoimmune diseases, we propose that iPSC-derived DCs could serve as a valuable tool for assessing IFN responses. Thus, to identify new therapeutic targets for AGS and interferonopathies, we conducted an extensive analysis of these iPSC-derived DCs with the *IFIH1* R779H mutation, particularly through RNA sequencing. The primary objective of this study is to discover new therapeutic targets and evaluate potential drugs for interferonopathies using an iPSC-based approach.

## Materials and Methods

### iPSCs

Blood samples were obtained from two sisters with SLE and two healthy female donors, from which we established iPSCs. Written informed consent was obtained from all subjects, and the experiments were conducted in accordance with the latest version of the Declaration of Helsinki. This study was approved by the Ethical Committee of the Institute of Medical Science at The University of Tokyo (2018153G). Healthy donor-derived iPSCs included TkC01 and PB002. TkC01 was established and provided by the Institute of Medical Science at the University of Tokyo from peripheral blood mononuclear cells of healthy females according to the protocol published in the Center for iPS Cell Research and Application (CiRA) [13]. PB002 was established and provided by Hideki Masaki, Institute of Medical Science at the University of Tokyo [14].

**Fig. 1:**
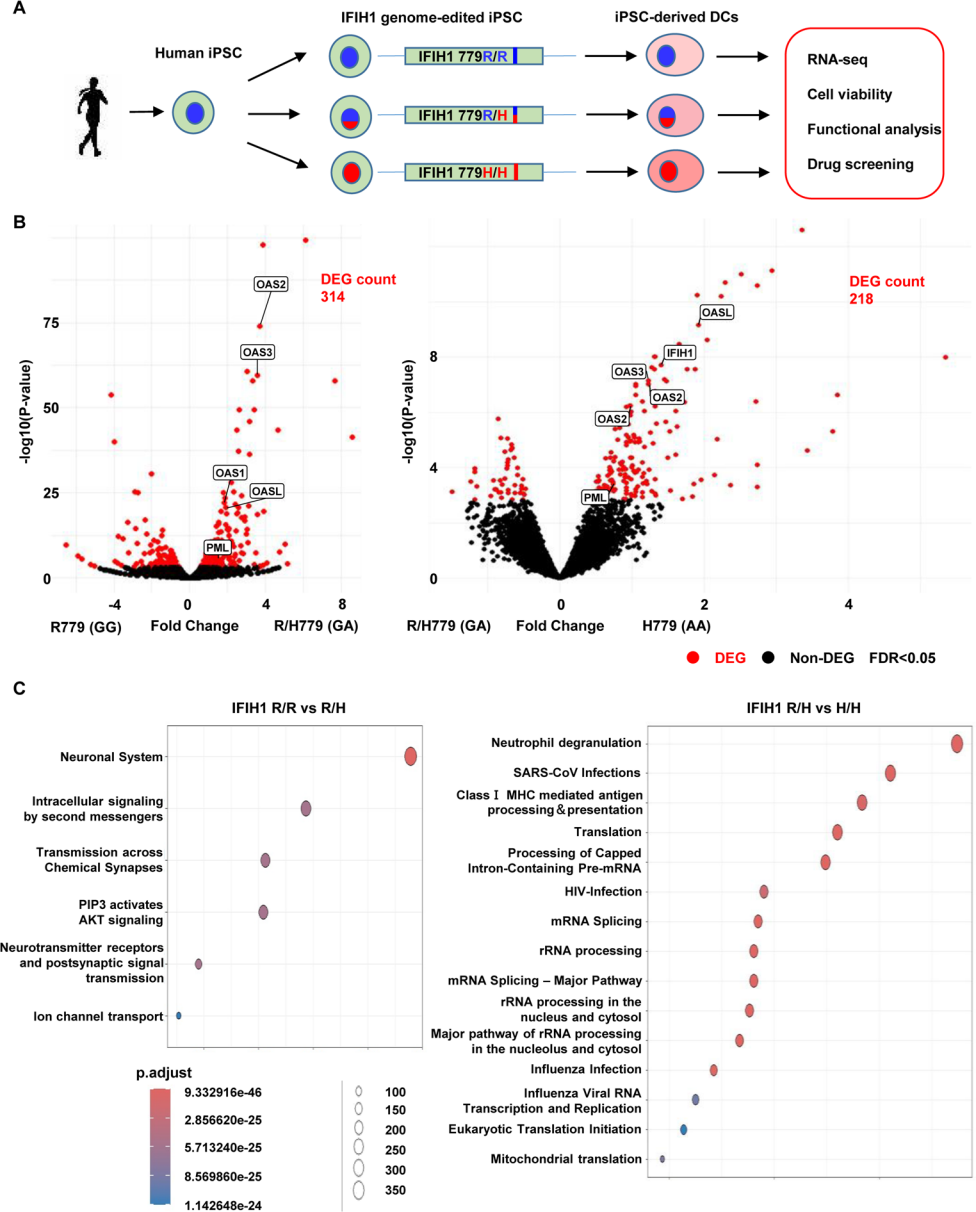
Study design and transcriptome analysis of iPSC-derived DCs **(A)** Schematic overview of the study design. iPSCs with *IFIH1* R779H mutation were generated through genome editing. IFN-α secretion was analyzed in iPSC-derived CD123^+^ DCs. **(B)** Volcano plots of differentially expressed genes (DEGs) in iPSC-derived CD123^+^ DCs with or without *IFIH1* R779H mutation. DEGs were indicated by red dots, with significance defined as a false discovery rate (FDR) < 0.05. (**C**) Pathway analysis of DEGs using ReactomePA. Top 15 pathways are indicated in dot plots

We previously established genome-edited iPSCs carrying either a heterozygous or homozygous *IFIH1* 779 H variant, as described in detail in our previous reports (Fig. 1A) [12]. In brief, genome editing was performed using our original footprint-free and seamless method. For transfection, we utilized a combination of a guide RNA expression vector (Addgene plasmid #41824) [15], the pSpCas9(BB)−2 A-GFP vector (PX458; Addgene plasmid #48138) [16], and a single-stranded oligodeoxynucleotide (ssODN) donor. Transfection was carried out using FuGENE HD (Promega). At 48 h post-transfection, GFP-positive cells were sorted, clonally expanded, and screened to identify successfully edited clones.

CD123^+^ dendritic cells (DCs) were differentiated from iPSCs following the protocol outlined in our prior study [12]. In brief, detached iPSC colonies were seeded on irradiated C3H10T1/2 cells (purchased from the Institute of Physical and Chemical Research Bio-Resource Center (Tsukuba, Ibaraki, Japan)) on day 1 in hematopoietic cell differentiation medium (Iscove’s modified Dulbecco’s medium supplemented with 15% fetal bovine serum (FBS), 1% penicillin streptomycin glutamine and a cocktail of 10 μg/mL human insulin, 5.5 μg/mL human transferrin, 5 ng/mL sodium selenite, 2 mmol/L l-glutamine, 0.45 mmol/L α-monothioglycerol, and 50 mg/mL ascorbic acid in the presence of 20 ng/mL VEGF). Other cytokines, including 50 ng/mL Flt-3 L (R&D Systems), 50 ng/mL SCF (R&D Systems) and 50 ng/mL TPO, were added on day 4. On day 14, hematopoietic progenitor cells (HPCs) were harvested from “sac-like” structures and seeded on newly irradiated C3H10T1/2 cells with differentiation medium [αMEM + 10% fetal bovine serum (Biological Industries, Kibbutz Beit Haemek, Israel) + 1% penicillin streptomycin glutamine] including 50 ng/ml Flt-3 L, SCF and TPO. We then performed a half medium change on day 17. On day 21 of differentiation, we harvested and counted floating and weakly adherent cells, then seeded 1.0 × 10⁵ cells per well into a 96-well plate pre-seeded with irradiated C3H10T1/2 feeder cells including 50 ng/ml Flt-3 L and TPO. Reagents for inhibitory analysis were added on day 23. On day 25, cells and culture supernatants were collected for downstream assays, including measurements of oxygen consumption rate (OCR), reactive oxygen species (ROS), cell viability, cytokine secretion, and phosphorylation levels. There were batch-to-batch variations in the differentiation efficiency into CD123⁺ DCs; therefore, only samples from batches with successful differentiation were used for further analysis. Compared to healthy iPSCs, SLE-derived iPSCs often exhibited higher differentiation efficiency into CD123⁺ DCs.

### Reagents

Anti-IFNAR inhibitory antibody (IFN-ARi) was obtained from Creative Biolabs (NAB-1570-VHH). Rapamycin (Rapa) was sourced from AdipoGen (AG-CN2-0025-M005). Mitochondrial complex inhibitors (MTCIs), including MT-I inhibitor (Rotenone) (13995), MT-II inhibitor (3-nitropropionic acid) (14684), and MT-V inhibitor (Oligomycin A) (19162), were purchased from Cayman Chemical [17, 18]. MT-IV inhibitor (Sodium Cyanide) was sourced from FUJIFILM (192-01852). Tofacitinib (Tofa) was purchased by Pfizer Japan, Baricitinib (Bari) by Eli Lilly Japan, and Trisenox (arsenic trioxide, ATO) by Nippon Shinyaku. In silico-predicted compounds, including bisantrene (BSM), phthalylsulfathiazole (PSZ), and ganaplacide (GNP), were purchased from Namiki Shoji Co., Ltd. Drug concentrations were optimized based on efficacy and cell viability. Final concentrations were: DMSO 0.01%, IgG2a 100 ng/mL, IFN-ARi 100 ng/mL, Tofa 20 ng/mL, Bari 50 ng/mL, MTCI-I (Rotenone) 100 nM, MTCI-II (3-nitropropionic acid) 100 µM, MTCI-IV (Cyanide) 1 µM, MTCI-V (Oligomycin) 100 nM, and Rapa 1 nM.

### RNA-Seq Library Preparation and Sequencing

Total RNA was extracted from iPSC-derived CD123^+^ DCs using the RNeasy Micro Kit (Qiagen), following the manufacturer’s protocol. RNA quality was verified using a 2100 Bioanalyzer (Agilent) and quantified using the Qubit RNA HS Assay Kit (Thermo Fisher Scientific). RNA-sequencing libraries were prepared with the SMART-Seq v4 Ultra Low Input RNA Kit (Illumina) according to the manufacturer’s instructions. Paired-end sequencing was performed with the MiSeq Reagent V4 kit (Illumina) on the MiSeq system. Sequencing data were converted from BCL to FASTQ format using bcl2fastq2 v2.20. Detailed sequencing, quality control, and mapping procedures were described in our previous study [19].

### Differentially Expressed Gene (DEG) Analysis

Genes with a read count of less than 10 in 90% or more of the samples within the same subset were excluded. Data normalization was performed using the R package TCC (version 1.22.0). Batch effects were removed using the R package RUVseq (version 3.24.0). Comparisons between groups were conducted using the quasi-likelihood F-test in edgeR (version 4.3.3). Genes with a false discovery rate (FDR) of less than 0.05 were considered significant. Pathway analysis of DEGs was performed using the R package ReactomePA [20].

### ELISA and SEAP Assay for Type I IFN Measurement

Type I IFN levels and bioactivity in culture supernatants were measured via ELISA and SEAP assay. The Human IFN-Alpha ELISA Kit (PBL Assay Science) was used following the manufacturer’s instructions. For the SEAP assay, HEK-Blue™ IFN-α/β reporter cells (InvivoGen) were employed to measure the bioactivity of IFN-α/β. A total of 20 µL of cultured cell supernatants was incubated with HEK-Blue™ IFN-α/β cells (5.0 × 10⁴ cells in 180 µL) for 24 h at 37 °C. Subsequently, 20 µL of the culture supernatant was added to 180 µL of QUANTI-Blue™ (InvivoGen) to quantify SEAP levels, which reflect the functional activity of type I IFNs in the samples.

### Reactive Oxygen Species (ROS) Measurement

Total ROS levels were assessed using the ROS Assay Kit-Highly Sensitive DCFH-DA (DOJINDO), following the manufacturer’s protocol. ROS levels were quantified by measuring the fluorescence intensity of oxidized 2’,7’-Dichlorodihydrofluorescein (DCF).

### Phosphorylated Interferon Regulating Factor 3 (IRF3) Measurement

Intracellular staining of phosphorylated IRF3 was conducted using the Intracellular Flow Cytometry Kit (Cell Signaling) according to the manufacturer’s instructions, and samples were analyzed using a FACS Aria II.

### Extracellular Flux Analysis

To examine the metabolic status, the oxygen consumption rate (OCR) and extracellular acidification rate were measured using the Seahorse XF 96 analyzer, following a standardized protocol [21]. iPSC-derived DCs (5.0 × 10^4^ cells/well) were plated in 96-well Seahorse plates coated with Cell-Tak (Corning). OCR, as an indicator of mitochondrial function, was measured in real-time, with cells sequentially treated with 1 µM oligomycin, 2 µM carbonyl cyanide-4-(trifluoromethoxy) phenylhydrazone (a mitochondrial uncoupler), and 0.5 µM rotenone plus 0.5 µM antimycin A (respiratory chain inhibitors).

### Cell Lines

Human monocytic cell line, THP-1 cells, were obtained from European Collection of Authenticated Cell Cultures (ECACC). These cells were cultured in RPMI1640, supplemented with 10% fetal bovine serum, 1 mM sodium pyruvate, and 100 nM penicillin/streptomycin. THP-1 cells were cultured at 5% CO2, 37 °C, at a low density (2.5 × 105/mL), with the media being refreshed every 3 days. To overexpress the OASL gene, lentiviral vectors (pLV-dTomato and pLV-dTomato-OASL) were constructed, and lentiviral particles were obtained from Vector Builder. Then it was introduced to THP-1 cells following the manufacturer’s protocol. Briefly, 5 × 10^5 cells were seeded in one well of a 24-well plate with 1 ml of medium. The virus, along with polybrene (5μg/ml), was added to the medium. The plate was gently rocked back and forth to mix. The plate was then centrifuged at 1000 x g for 1 h at 32 °C. After removing the virus-containing medium, 1 ml of fresh complete growth medium was added. Cells were carefully resuspended by pipetting up and down and incubated in the culture incubator. Forty-eight hours after lentiviral transduction, dTomato-high cells were sorted by FACS and expanded. THP-1 cells (5 × 10⁵), either overexpressing dTomato (control) or OASL, were stimulated with 3 µg/mL of low molecular weight poly(I: C) (LeoVec, InvivoGen), followed by treatment with various compounds. LeoVec, a transfection reagent, delivers ligands to the cytosol, and Poly (I: C) LMW stimulates cytosolic receptors. IFN-alpha levels were measured using the SEAP assay, and the efficacy concentration for 50% inhibition (EC50) was calculated. Cell viability was assessed with the Cell Counting Kit-8 (DOJINDO), and the cytotoxic IC50 was determined. The therapeutic index (TI) was calculated as the ratio of cytotoxic IC50 to EC50, with candidates selected based on a TI greater than 3, in line with criteria for narrow TI drugs [22].

### In Silico Prediction

The drug library from the ChEMBL database (ChEMBL29; 10,409 compounds) [23] was downloaded, and counterions and inorganic compounds were removed using MayaChemTools (2021) [24]. 3D structures were generated using Balloon (version 1.8.1) [25] with an MMFF94 force field, and protonation states were adjusted to pH 7.4 using Babel (version 2.4.1) [26]. A total of 9,597 compounds underwent virtual screening using rDock (2013) [27]. We used the crystal structure of the human OASL protein (PDB ID: 4XQ7), focusing on the RNA-binding groove of its OAS-like domain. Before simulations, dsRNA was removed from the OLD structure [12], and the rDock cavity was defined using the two-sphere method. Simulations produced 50 docking models per compound, yielding 475,150 docking modes. Compounds with an rDock score ≤ − 50 kcal·mol^−1 were selected, resulting in 83 compounds (Supplementary Table S1). The ChEMBL IDs of these compounds were processed using KNIME (version 4.1.2) [28] to retrieve relevant data.

### Statistical Analysis

GraphPad Prism 7.02 was used for all statistical analyses, except for sequence data analysis. For comparisons involving more than three groups, ANOVA with Tukey’s Honestly Significant Difference test or the Kruskal-Wallis test with Dunn’s multiple comparisons test was applied. Correlations were evaluated using Spearman’s rank correlation. P-values < 0.05 were considered statistically significant.

## Results

### Transcriptome Analysis of CD123^+^ DCs with IFIH1 R779H Mutation

In a recent study, we successfully established iPSCs with an *IFIH1* R779H mutation using genome editing techniques for single nucleotide replacement (Supplementary Figure S1) [12]. The *IFIH1* R779H mutation is known to cause Aicardi-Goutières syndrome (AGS) [3, 7]. This mutation led to spontaneous secretion of IFN-α from iPSC-derived CD123^+^ dendritic cells (DCs), with secretion levels further increasing in response to cytosolic dsRNA, a ligand for the MDA5 receptor [12]. Our current objective was to identify potential therapeutic targets for AGS by performing disease modeling and drug investigation on these *IFIH1*-mutated iPSC-derived DCs (Fig. 1A). Transcriptome analysis revealed hundreds of DEGs when comparing wild-type and *IFIH1* R779H heterozygous/homozygous iPSC-derived CD123^+^ DCs (Fig. 1B). Notably, 84 DEGs showed increased expression in an *IFIH1* R779H dose-dependent manner (Supplementary Figure S2). These DEGs included several IFN-related genes, such as members of the oligoadenylate synthetase (OAS) family. Pathway analysis highlighted the upregulation of IFN signaling and anti-viral mechanism pathways, closely associated with *IFIH1* function (Fig. 1C). Additionally, pathways related to ribosome and transcription were upregulated, suggesting activation of protein synthesis in *IFIH1*-mutated iPSC-derived DCs. Notably, a pathway associated with mitochondrial translation was also upregulated, suggesting alteration of mitochondrial functions. This led to the hypothesis that targeting mitochondria could be a potential therapeutic approach for AGS.

### Mitochondrial Activation in CD123^+^ DCs Derived from iPSCs with IFIH1 Mutation

**Fig. 2:**
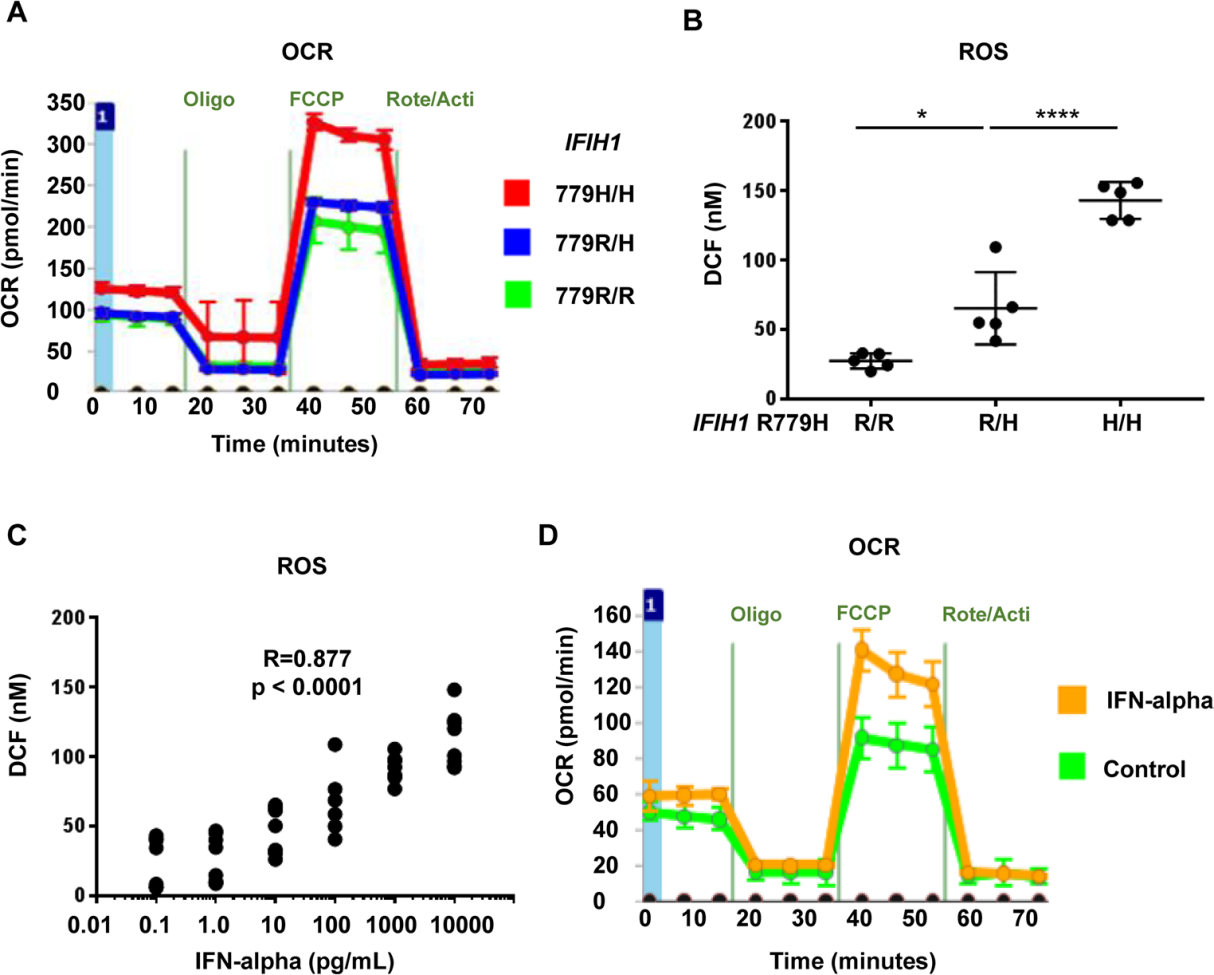
Mitochondria activation in iPSC-derived CD123^+^ DCs **(A)** Oxygen consumption rate (OCR) of iPSCs under the DC differentiation protocol, measured by flux analyzer. **(B)** Mitochondrial reactive oxygen species (ROS) levels in culture supernatant, assessed by the fluorescent intensity of oxidated 2’, 7’-Dichlorodihydrofluorescein (DCF). ANOVA and Tukey’s honestly significant difference test. *p < 0.05; ***p < 0.001. **(C)** Correlation between IFN-α levels and ROS in the supernatants of iPSCs under the DC differentiation protocol. Unedited (wild-type) iPSCs were differentiated into CD123⁺ DCs, and IFN-α was added on day 23, as illustrated. Culture supernatants were collected on day 25, and ROS levels were measured. Spearman’s rank correlation (*p* < 0.0001, *R* = 0.877). **(D)** OCR in exogenous IFN-α stimulated wild-type iPSCs under the DC differentiation protocol, measured by flux analyzer. Results were validated in three or more independent experiments

**Fig. 3:**
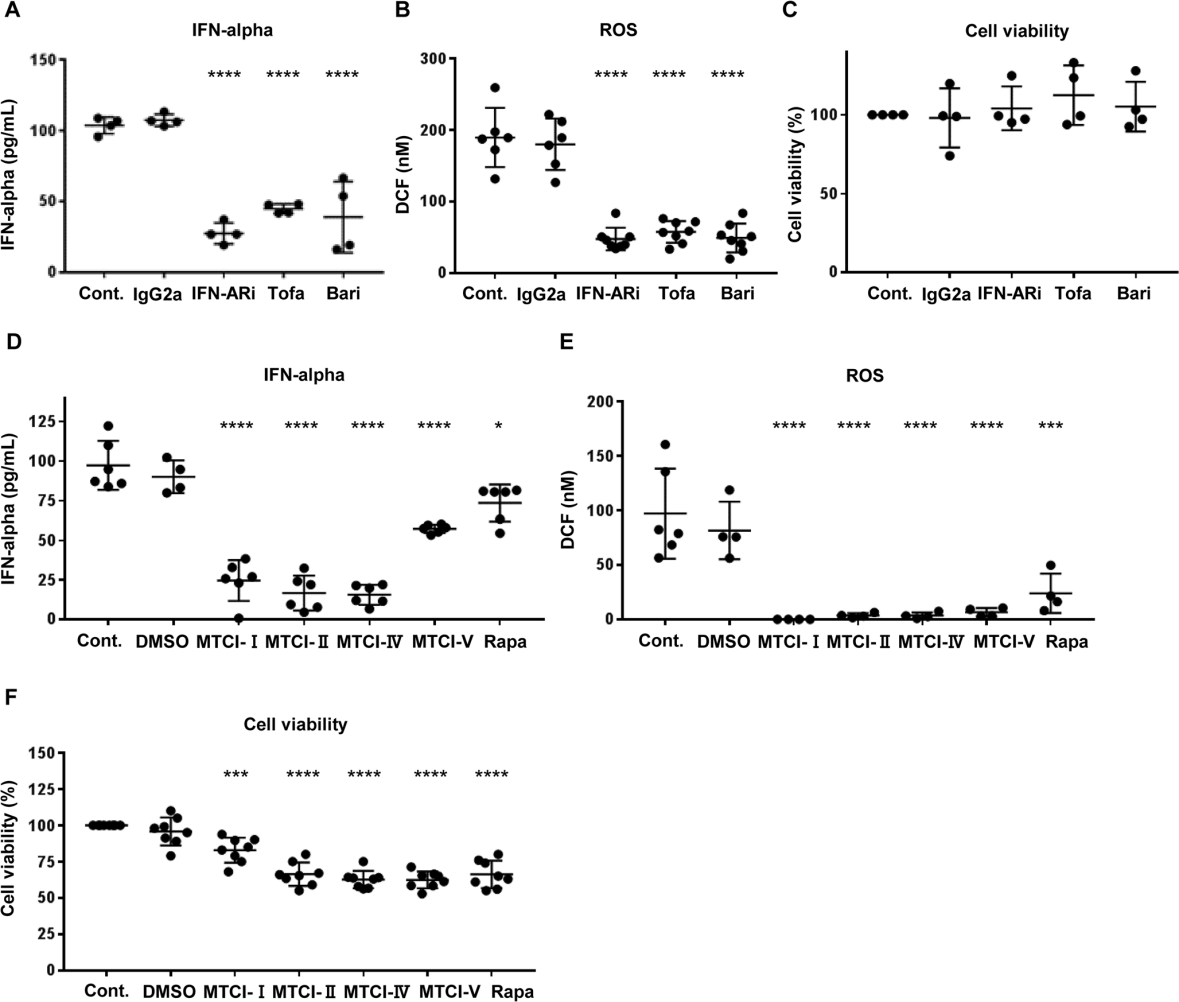
Suppression of IFN-α secretion in iPSC-derived CD123^+^ DCs with *IFIH1* 779 H/H mutation **(A**,** D)** IFN-α concentrations in supernatant of iPSC-derived CD123^+^ DCs with *IFIH1* 779 H/H mutation with drug concentrations based on previous reports and therapeutic index (TI) (see Methods). **(B**,** E)** ROS concentration in culture supernatants, measured by the fluorescent intensity of oxidized 2’, 7’-DCF. **(C**,** F)** Cell viability under drug conditions in iPSC-derived CD123^+^ DCs with *IFIH1* 779 H/H mutation, assessed by flow cytometry (PI stain). Kruskal-Wallis test with Dunn’s multiple comparisons test, with test samples compared to isotype controls (IgG2a). * *p* < 0.05; ***p < 0.001; ****p < 0.0001. Cont.: control, IFN-ARi, Interferon receptor alpha inhibitory antibody. Tofa: tofacitinib, Bari: baricitinib. MTCI: mitochondrial complex inhibitor, Rapa: rapamycin. Results were validated in three or more independent experiments

Further analysis focused on mitochondrial activity in *IFIH1* R779H-mutated iPSC-derived CD123^+^ DCs. Oxygen consumption rate (OCR), an indicator of mitochondrial activity, was significantly elevated in *IFIH1* R779H-mutated DCs (Fig. 2A), accompanied by increased total cellular reactive oxygen species (ROS) production (Fig. 2B). It is important to note that in Fig. 2A and 2B, metabolic activity and ROS levels were altered by the variant itself, without the addition of IFN or any other external stimulation. Importantly, a positive correlation was observed between mitochondrial ROS and IFN-α secretion in the *IFIH1* R779H-mutated DCs (Fig. 2C). Additionally, exogenous IFN-α stimulation resulted in increased OCR (Fig. 2D). Blocking IFN signaling with anti-IFNAR inhibitory antibodies (IFN-ARi) or JAK inhibitors suppressed both IFN-α secretion and mitochondrial ROS production, while maintaining cell viability (Fig. 3A-C). These results indicated that IFN signaling plays a key role in mitochondrial activation in *IFIH1* R779H-mutated DCs.

### Regulating Mitochondrial Activity Alleviated Excessive IFN-α Secretion from IFIH1 R779H-Mutated iPSC-Derived CD123^+^DCs

To test whether regulating mitochondrial activity could reduce excessive IFN-α secretion in *IFIH1* R779H-mutated DCs, we applied mitochondrial complex inhibitors (MTCIs) that target various components of the oxidative phosphorylation (OXPHOS) pathway [17, 18]. MTCIs effectively decreased both IFN-α secretion and ROS production in *IFIH1* R779H-mutated DCs (Fig. 3D, E). Regulation of the mTOR pathway using rapamycin (Rapa) also resulted in reduced IFN-α secretion and ROS production (Fig. 3D, E), but with a narrow therapeutic index and increased cytotoxicity (Fig. 3F). These findings suggest that mitochondrial metabolism is a promising therapeutic target for interferonopathies, although cytotoxicity remains a concern for clinical application.

#### Drug Repositioning Candidates for Novel Treatment of Type I Interferonopathy

**Fig. 4:**
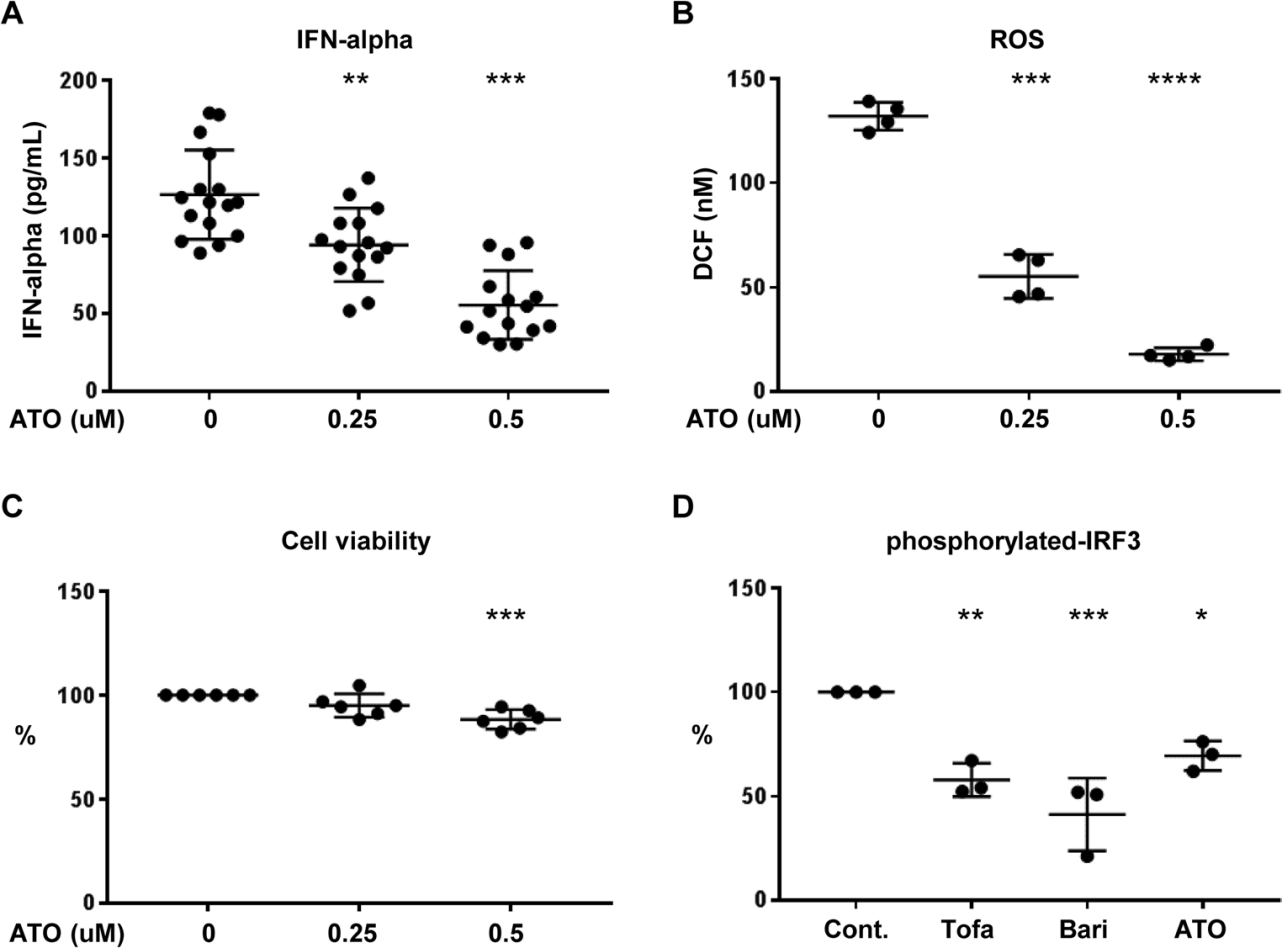
Arsenic trioxide (ATO) suppression test in iPSC-derived DCs with *IFIH1* 779 H/H mutation **(A)** IFN-α concentrations in supernatant of iPSC-derived CD123^+^ DCs with *IFIH1* 779 H/H mutation in the presence of ATO. **(B)** ROS concentrations in supernatants of iPSC-derived CD123^+^ DCs with *IFIH1* 779 H/H mutation, measured by the fluorescent intensity of oxidized 2’, 7’-DCF. **(C)** Cell viability under the drug conditions in iPSC-derived CD123^+^ DCs with *IFIH1* 779 H/H mutation, measured by PI staining. **(D)** Frequencies of intracellular phosphorylated-IRF3-positive cells in iPSC-derived CD123^+^ DCs with *IFIH1* 779 H/H mutation. Tofa: tofacitinib, Bari: baricitinib. Test samples were compared to controls. Kruskal-Wallis test with Dunn’s multiple comparisons test. *p < 0.05; **p < 0.01; ***p < 0.001; ****p < 0.0001. Results were validated in three or more independent experiments

To identify therapeutic candidates for type I interferonopathy, we explored genes of interest from the DEG list of *IFIH1* R779H-mutated DCs (Fig. 1B; Supplementary Figure S2). Drug repositioning was considered a viable strategy due to its potential to expedite the drug development process. We focused on the upregulated PML (Promyelocytic leukemia protein) gene, because its expression correlated with the *IFIH1* R779H mutation (Fig. 1B; Supplementary Figure S2) and effective drugs targeting PML are already available. PML is targeted by arsenic trioxide (ATO), a drug approved for acute leukemia [29]. ATO is currently undergoing phase Ⅱ clinical trials for SLE [30]. ATO significantly suppressed IFN-α secretion and reduced ROS production in *IFIH1* R779H-mutated DCs, with minimal cytotoxicity observed (Fig. 4A-C). Additionally, ATO, similar to JAK inhibitors, inhibited phosphorylation of interferon regulatory factor 3 (IRF3), a key step in IFN signaling (Fig. 4D). These findings suggest ATO as a promising therapeutic candidate for AGS and interferonopathies.

#### Drug Screening Targeting OAS Family Molecules for Type I Interferonopathy

**Fig. 5:**
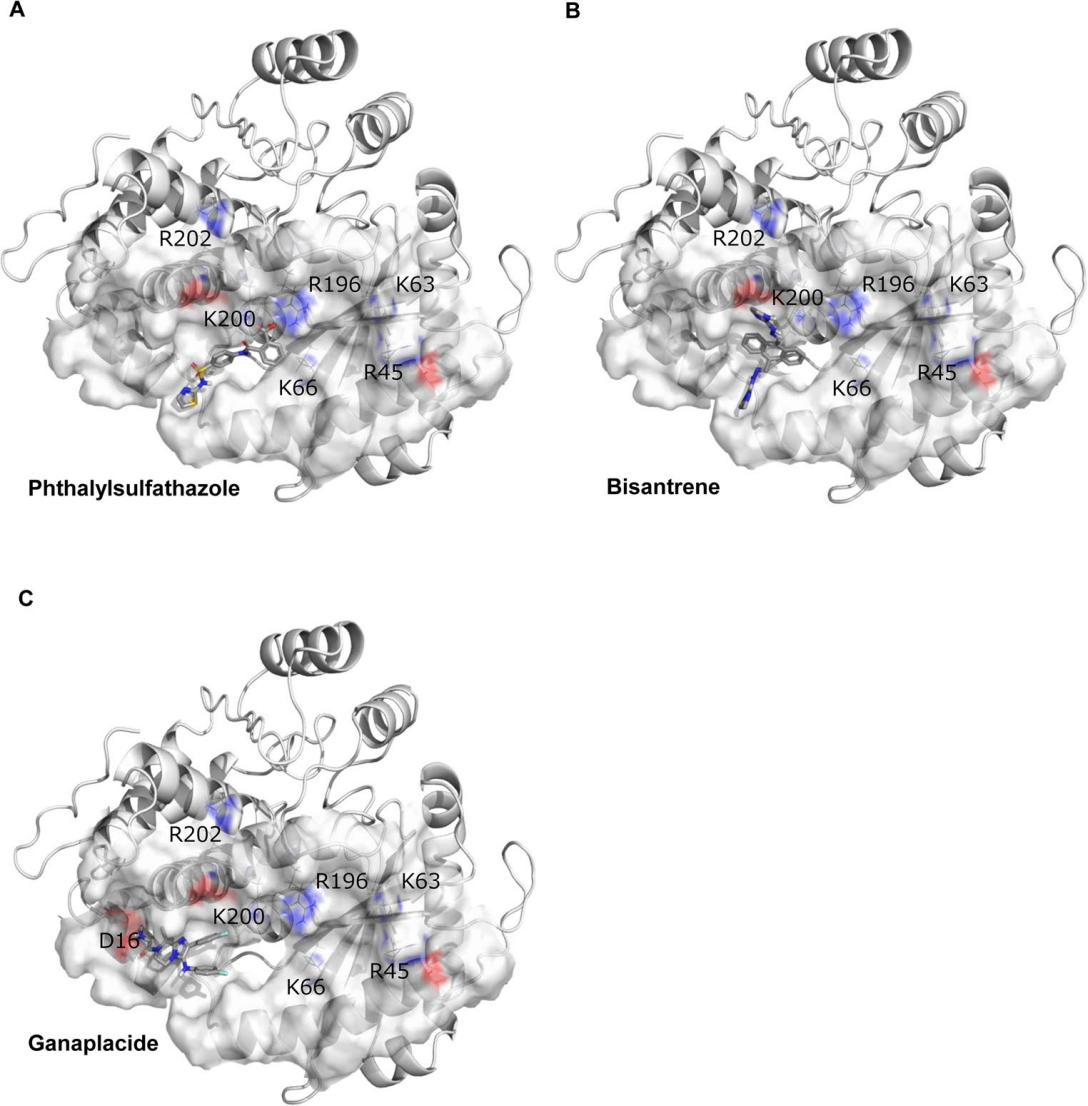
In silico prediction of compounds bound to the RNA-binding groove of the OAS-like domain (OLD) Structures of hOLD with compounds bound in the RNA-binding groove. Key residues at the dsRNA-binding groove are shown in CPK coloring. Compounds illustrated: (**A**) Phthalylsulfathiazole (PSZ), (**B**) Bisantrene (BSA), (**C**) Ganaplacide hydrochloride (GNP)

**Fig. 6:**
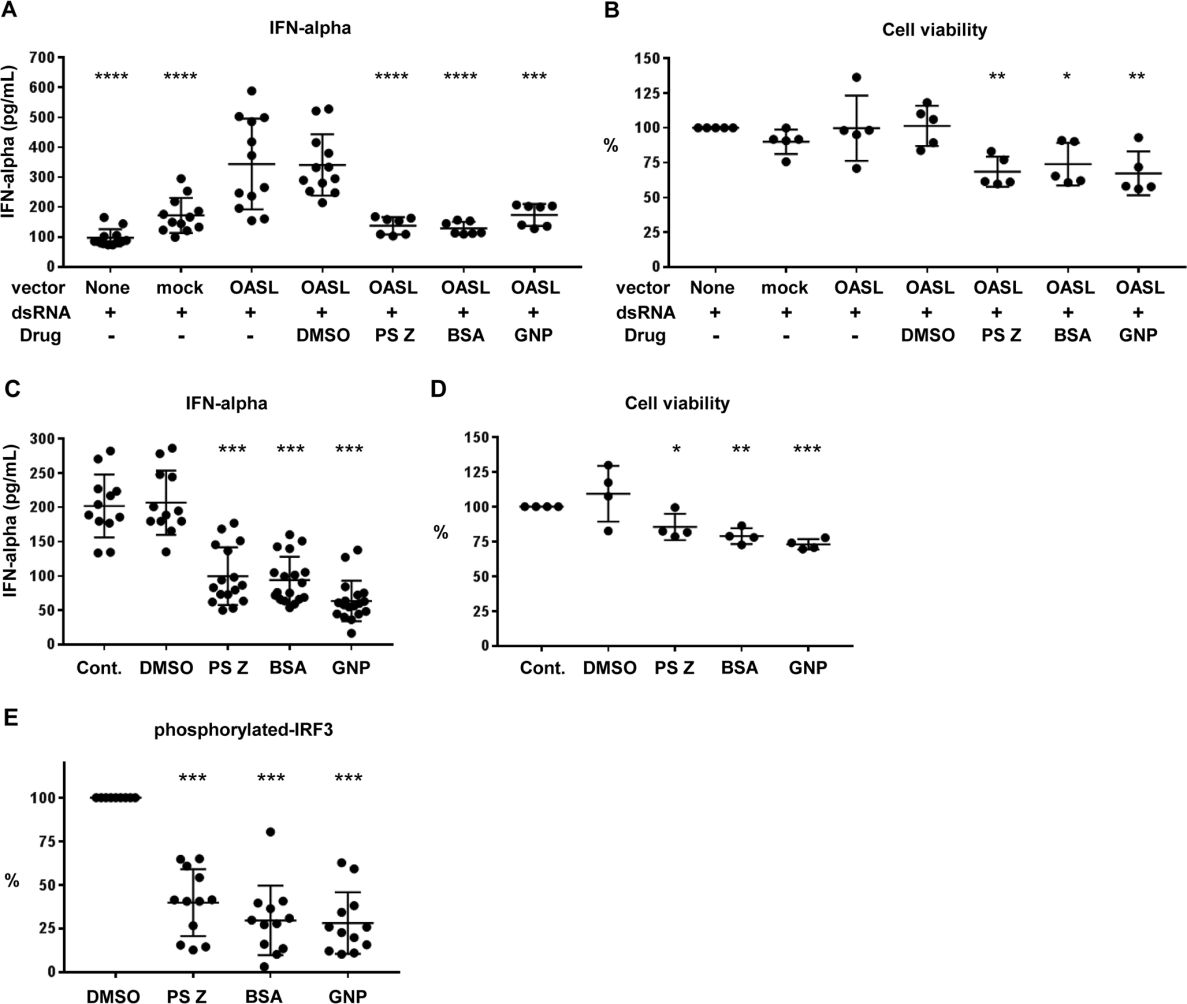
Suppression tests with OLD-binding compounds in OASL-transfected cells and iPSC-derived DCs.**(A**) IFN-α concentrations in supernatant of OASL-transfected THP-1 cells in the presence of 3 μg/mL LeoVec Poly (I: C) LMW and the predicted OAS-binding compounds. Compound concentrations: Phthalylsulfathiazole (PSZ) 250 μM, Bisantrene (BSA) 250 nM, Ganaplacide hydrochloride (GNP) 1 nM. **(B)** Cell viability of OASL-transfected cells measured by PI staining. **(C)** Supernatant concentrations of IFN-alpha in iPSC-derived CD123^+^ DCs with IFIH1 779 H/H mutation in the presence of the predicted OAS-binding compounds. **(D)** Cell viability under the drug concentrations of iPSC-derived CD123^+^ DCs with *IFIH1* 779 H/H mutation was measured by PI staining. **(E) **Frequencies of intracellular phosphorylated-IRF3-positive cells in iPSC-derived CD123^+^ DCs with *IFIH1* 779 H/H mutation. Comparisons to DMSO controls were made. Kruskal-Wallis test with Dunn’s multiple comparisons test. *p < 0.05; **p < 0.01; ***p < 0.001; ****p < 0.0001. Results were validated in three or more independent experiments

Next, we focused on the upregulated OAS-like (OASL) gene, whose expression correlated with the IFIH1 R779H mutation (Fig. 1B; Supplementary Figure S2), as it represents a molecule that we have been investigating in our ongoing research [12]. The OAS family genes, including OAS1, OAS2, OAS3, and OASL, were significantly upregulated in *IFIH1* R779H-mutated DCs (Fig. 1B; Supplementary Figure S2). OASL-deficiency suppressed IFN-α secretion in response to the *IFIH1* R779H mutation, suggesting its critical role in *IFIH1*-induced IFN-α secretion [12]. Consequently, OAS molecules became promising targets for potential drug interventions. To explore new drug candidates targeting OAS family molecules, we conducted structure-based virtual screenings of compounds that could bind to, and possibly seal, the RNA-binding groove in OAS-like domain (OLD) of human OASL, using relatively reliable rDock docking program (Fig. 5; Supplementary Table S1). We identified 83 compounds with lower, rDock scores (Supplementary Table S1) from drug library of ChEMB29 database (10,409 compounds), and 7 compounds were selected for further analysis based on availability and human-use status while excluding compounds with well-known targets, such as kinase inhibitors or receptor blockers. In OASL-transfected THP-1 cells, three compounds, ganaplacide hydrochloride (GNP), bisantrene (BSA), and phthalylsulfathiazole (PSZ), demonstrated a therapeutic index (TI) above the threshold of three, indicating promising efficacy and low toxicity (Figs. 5A-C and 6A and B; Supplementary Table S2). These compounds targeted RNA-binding regions in the OLD, masking crucial residues for RNA interaction. GNP, BSA, and PSZ effectively suppressed IFN-α secretion in *IFIH1* R779H-mutated DCs with low cytotoxicity (Fig. 6C, D) and significantly reduced IRF3 phosphorylation (Fig. 6E). Thus, our iPSC-based disease modeling and drug investigation strategy, complemented by in silico predictions, has successfully identified new therapeutic targets for interferonopathies.

## Discussion

Interferonopathies represent a group of severe infantile autoinflammatory diseases for which effective therapies remain limited [1, 2]. iPSC-based drug screening offers a promising approach to addressing these challenges. For instance, Nakajo-Nishimura syndrome (NNS), one of the interferonopathies caused by mutations in PSMB8, was previously studied using iPSC-based drug screening systems derived from NNS patients [11]. Such strategies hold promise, particularly for rare genetic diseases. In prior research, we introduced causal mutations into iPSCs via genome editing and established a differentiation protocol for generating iPSC-derived CD123^+^ DCs that secrete IFN-α in response to dsRNA stimulation [12]. Here, we propose that *IFIH1* R779H-mutated iPSC-derived CD123^+^ DCs can serve as a valuable tool for drug investigation in the context of interferonopathies, particularly AGS and SLE. iPSC-based disease modeling offers the advantage of enabling the consistent use of human cells, including rare populations like DCs, under controlled conditions, as well as simplifying in vitro drug investigation tests [9]. Our identification of PML as a candidate gene regulating interferonopathies through transcriptome analysis enabled us to swiftly conduct inhibitory experiments using its antagonist, ATO [29]. Additionally, in silico predictions facilitated drug screening for interferonopathies by identifying compounds that bind to OAS and inhibit IFN-α secretion. Overall, iPSC-based disease modeling holds significant promise for identifying new therapeutic targets, particularly for rare diseases.

In this study, we investigated the mechanisms underlying excessive IFN-α production driven by the *IFIH1* R779H mutation, highlighting the crucial role of mitochondrial activation. *IFIH1* R779H-mutated iPSC-derived CD123^+^ DCs exhibited excessive IFN-α secretion, even in the absence of exogenous stimulation. Previous studies have suggested that endogenous dsRNA serves as a stimulatory source for *IFIH1*-dependent pathways [7]. This includes mitochondria-derived dsRNA (mtRNA), which recent reports show can stimulate *IFIH1* [31]. Interestingly, anti-mtRNA antibodies have been detected in SLE patients [32]. Mitochondrial activation in *IFIH1* R779H-mutated DCs likely promotes an excess release of mtRNA. Furthermore, mitochondrial antiviral signaling (MAVS) on the mitochondrial membrane facilitates *IFIH1* signaling, while mitochondrial dynamics and ROS generation are essential for IFN signaling [33, 34]. In *IFIH1* R779H-mutated DCs, mitochondrial activation depends on IFN-α and JAK signaling, whereas IFN-α secretion is reliant on mitochondrial oxidative phosphorylation (OXPHOS). We propose that a positive feedback loop may drive excessive IFN-α secretion in AGS patients, suggesting that interrupting this feedback loop could be a potent therapeutic approach. In addition, our results that MTCIs suppressed both IFN-α and ROS production supports previous evidence suggesting that mitochondrial activation occurs downstream of IFIH1 activation and upstream of IFN-α production. *IFIH1* activation induces intrinsic mitochondrial activation, then enhanced IFN signaling. In this model, mitochondrial membrane potential, fusion dynamics, and ROS generation intrinsically amplify Retinoic acid-inducible gene I–like receptor (RLR) signaling [35]. Regulating mitochondrial activity in DCs, as identified via iPSC-based disease modeling, may offer a novel therapeutic strategy.

We particularly focused on DEGs, such as PML, as potential therapeutic targets. The availability of approved inhibitors for these targets makes them promising candidates for drug repositioning. PML is a nuclear protein that contributes to IFN signaling by forming nuclear protein complexes known as PML-nuclear bodies, which enhance gene expression associated with IFN responses, antiviral defense, and apoptosis [36, 37]. The chimeric gene PML-RARα, responsible for acute promyelocytic leukemia (APL), is targeted by ATO, an established therapy for APL [29]. Notably, ATO has shown some clinical efficacy in a Phase Ⅱa trial for SLE [30]. OAS family molecules are RNA-binding proteins that play critical roles in processing cytosolic RNA and inducing IFN responses [38–40]. As key IFN signature genes, OAS family molecules represent potential new therapeutic targets for interferonopathies. Here, we identified three compounds that bind to OAS proteins and inhibit IFN secretion: PSZ, GNP, and BSA. PSZ is a classic sulfa drug used to treat inflammatory conditions such as rheumatoid arthritis and inflammatory bowel disease [41, 42]. GNP, an imidazolopiperazine compound, is a newly developed anti-malarial drug [43]. BSA has anthracycline-like antineoplastic activity with reduced cardiotoxicity, currently undergoing Phase 2 trials for leukemia [44]. Although these drugs have not been previously reported to exhibit anti-inflammatory activity, they may inhibit IRF3 activation downstream of OAS and cytosolic RNA receptors [45]. Thus, these compounds likely interfere with IFN-related pathways, though further studies are needed to elucidate their precise mechanisms. These findings demonstrate the potential of iPSC-based disease modeling for addressing the clinical challenges of intractable diseases through drug repositioning.

Our study has limitations, notably the use of iPSC-derived cells without in vivo experiments, as these were outside the scope of this study. Although *IFIH1*-mutated model mice have been reported, pre-clinical trials with identified drug candidates are warranted in future studies. Additionally, we did not utilize primary patient cells, which presents a limitation given the challenges in obtaining sufficient samples from patients with rare diseases. However, our iPSC-based approach partially addresses this issue. Total cellular reactive oxygen species (ROS) were assessed using DCFDA, which detects total cellular ROS but does not specifically distinguish mitochondrial ROS. The role of PML was assessed using ATO, which has pleiotropic effects on cellular function. Although our results suggest that targeting PML may suppress aberrant IFN-α production, further studies using more specific approaches such as siRNA-mediated knockdown will be necessary to validate the contribution of PML to the pathogenesis of AGS.

For drug investigation, we established a TI threshold of greater than three [22], although a TI greater than ten is typically considered safe. Given the rarity and severity of interferonopathies, with no established standard therapies, a narrower TI may be acceptable when developing new therapeutic options.

## Conclusions

Our study elucidated the pathogenesis of interferonopathies and identified new therapeutic targets using *IFIH1* R779H-mutated iPSC-derived CD123^+^ DCs. This iPSC-based approach highlights the potential of mitochondrial metabolism and several DEGs as promising therapeutic candidates for interferonopathies. Drug repositioning provides a promising pathway for developing new treatments for rare diseases lacking effective therapies, such as AGS. Therefore, we conclude that iPSC-based strategies represent a powerful tool for analyzing and validating the efficacy and toxicity of candidate therapeutic agents for rare and intractable human diseases.

## Supplementary Information

Below is the link to the electronic supplementary material.

Supplementary Material 1

## Data Availability

No datasets were generated or analysed during the current study.
