# Supplementary material for: Identification of Potential Therapeutic Agents for Type I Interferonopathy Using iPSC-Based Disease Modeling
Source: J Clin Immunol. 2025 Sep 30;45(1):140. doi: 10.1007/s10875-025-01933-8 (PMC12484275; doi:10.1007/s10875-025-01933-8)
Supplement: Supplementary file 1 — Supplementary Material 1 [file 10875_2025_1933_MOESM1_ESM.docx]

**Supplementary information**

**Supplementary Figure S1.**

Schematic overview of IFIH1 R779H genome editing in iPSCs. Detailed experimental procedures are provided in reference 12.


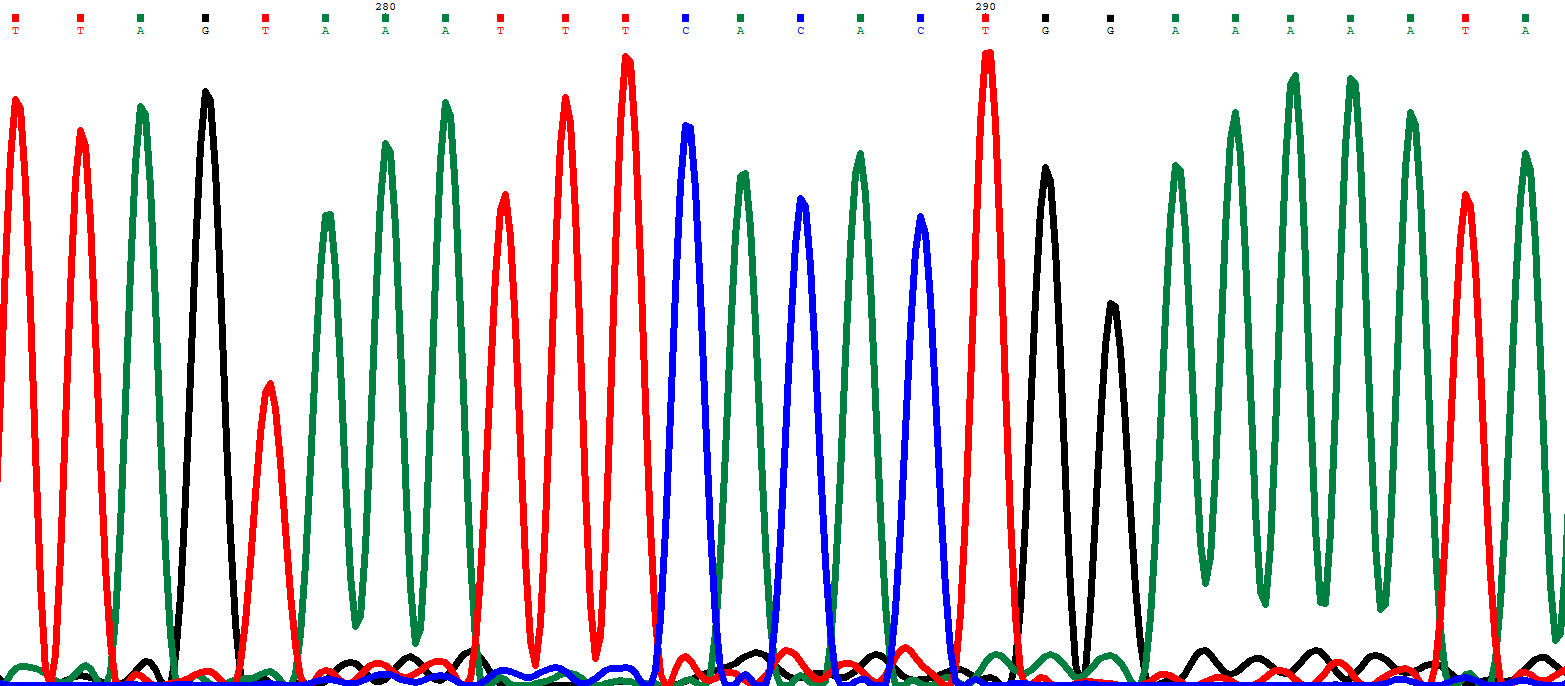


**IFIH1 779R / 779H**

**IFIH1 779H**

**T T T CA C A C T G**

**Variant (A/A)**

**Variant (G/A)**

**IFIH1 779R**

**Wild type (G/G)**

**T T T CG/AC A C T G**

**T T T CG C A C T G**


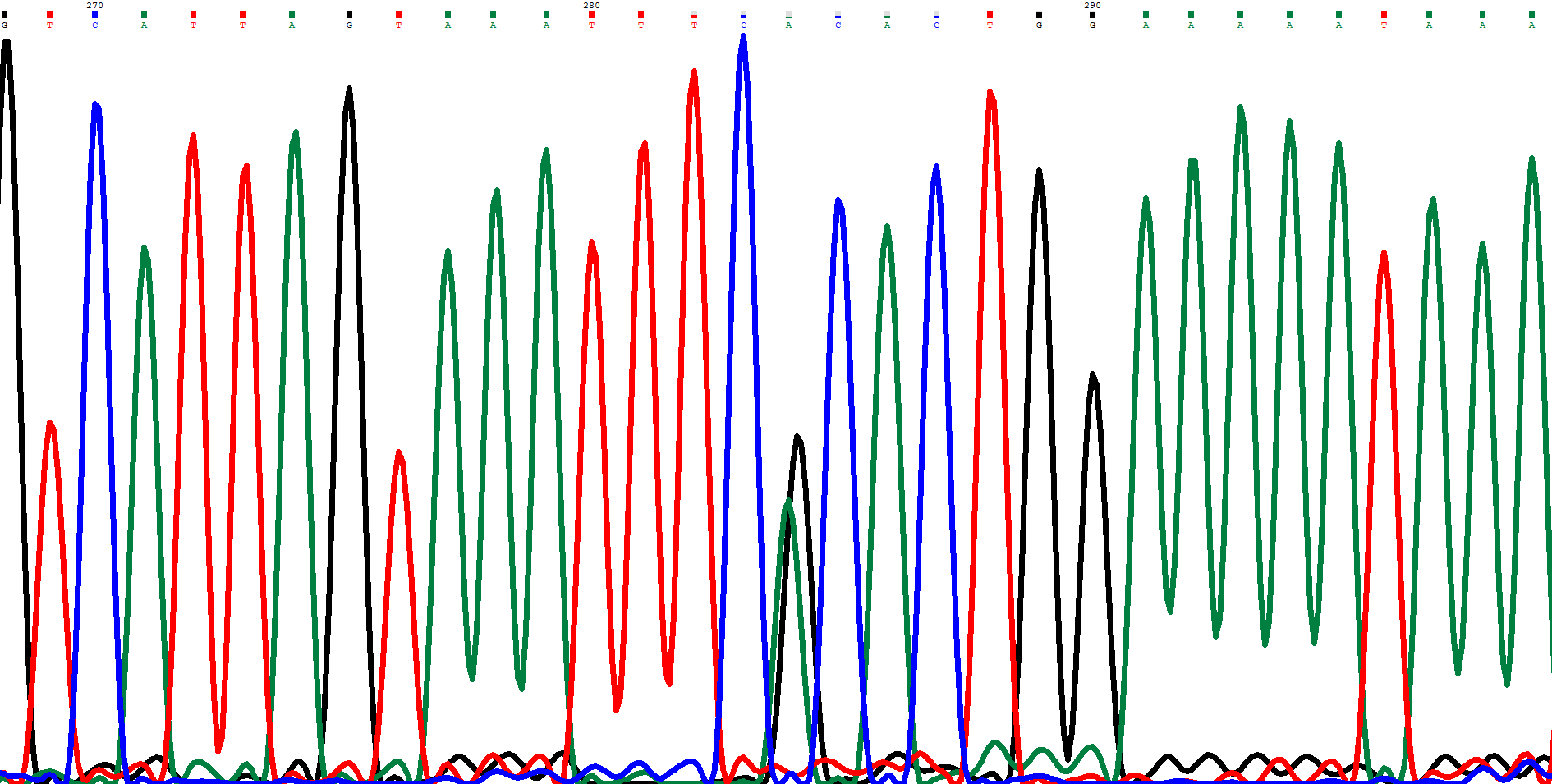

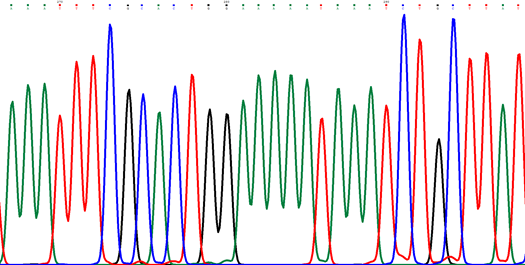

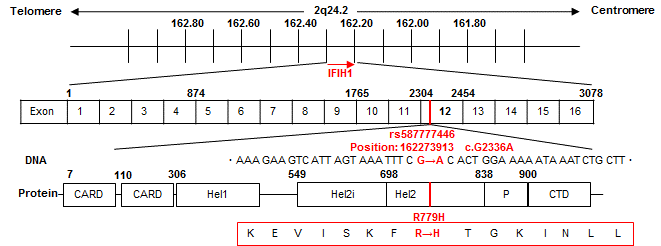


**Supplementary Figure S2. Heatmap of DEGs between iPSC-derived CD123^+^DCs with or without *IFIH1* R779H mutation.**


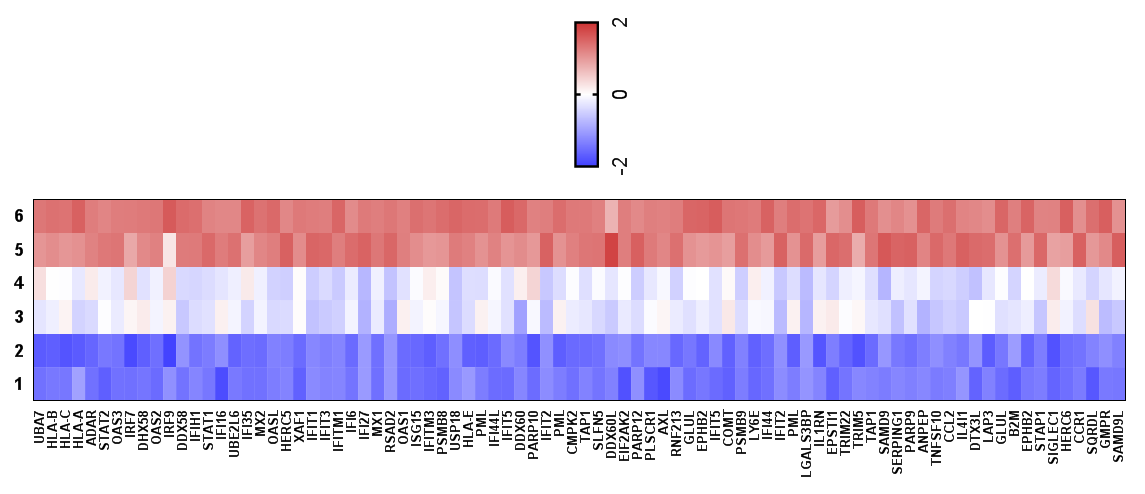

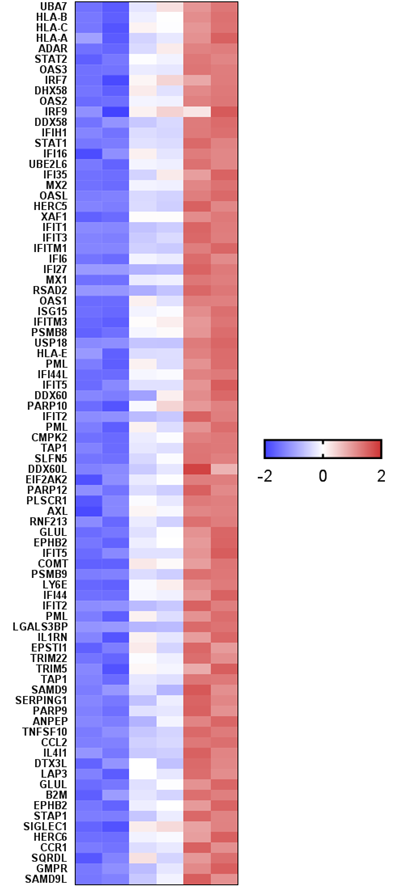


**R/R**

**1**

**R/R**

**2**

**R/H**

**1**

**R/H**

**2**

**H/H**

**1**

**H/H**

**2**

***IFIH1***

RNA-seq in iPSC-derived CD123^+^DCs with of wild type (IFIH1 R/R) and IFIH1 779 R/H and H/H revealed 84 variant dose dependently upregulated genes.

**Supplementary Table S1**. rDock scores of OLD-binding candidate compounds. Eighty-three compounds were predicted to bind OAS-like domain (rDOCK score < -50 kcal/ml). Among them, 13 compounds were approved and 31 were applied to clinical trial.

| ChEMBL ID | Name | Synonym | Phase | Action | Target | rDOCK score (kcal/mol) |
| --- | --- | --- | --- | --- | --- | --- |
| CHEMBL295409 | TIAMENIDINE | HOE 440 |  | Anti-hypertensive | Oryctolagus cuniculus | -85.4786 |
| CHEMBL297884 | CILUPREVIR | BILN-2061 | 2 |  |  | -72.405 |
| CHEMBL2105398 | SULFAMETROLE | Sulfametrole |  |  |  | -68.946 |
| CHEMBL2104704 | NOBERASTINE | Noberastine |  | Antihistaminic |  | -68.355 |
| CHEMBL1421 | DASATINIB | BMS-354825 | Approved |  | T cells | -66.875 |
| CHEMBL2104945 | SULFACLORAZOLE | Sulfaclorazole |  |  |  | -66.041 |
| CHEMBL450117 | GLYPROTHIAZOL | Glyprothiazol |  |  | Monoamine oxidase A | -65.851 |
| CHEMBL2106807 | MALEYLSULFATHIAZOLE | Maleylsulfathiazole |  |  |  | -65.210 |
| CHEMBL2105584 | ULDAZEPAM | U-31,920 |  | Sedative-Hypnotic |  | -64.885 |
| CHEMBL593262 | PARA-NITROSULFATHIAZOLE | Nisulfazole |  |  | Leishmania infantum | -62.592 |
| CHEMBL1015 | EVANS BLUE | AZOVAN BLUE | Approved |  | Menin/Histone-lysine N-methyltransferase MLL | -61.919 |
| CHEMBL1481457 | GLYSOBUZOLE | Glysobuzole |  |  | Aldehyde dehydrogenase 1A1 | -61.863 |
| CHEMBL2104561 | ELTENAC | Eltenac |  |  |  | -61.047 |
| CHEMBL1524273 | **PHTHALYLSULFATHIAZOLE** | Phthalylsulfathiazole | Approved |  | Cytochrome P450 3A4 | -60.704 |
| CHEMBL2110765 | BATELAPINE | Batelapine |  | Antipsychotic |  | -59.462 |
| CHEMBL2058833 | **GANAPLACIDE** | Ganaplacide | 2 |  | Plasmodium berghei | -59.113 |
| CHEMBL2106914 | PHTHALYLSULFAMETHIZOLE | Phthalylsulfamethizole |  |  |  | -58.791 |
| CHEMBL2104022 | ACLANTATE | Aclantate |  |  |  | -55.714 |
| CHEMBL2105025 | IQUINDAMINE | Iquindamine |  |  |  | -55.090 |
| CHEMBL3989868 | TUCATINIB | ARRY-380 | Approved |  | Adaptor-associated kinase | -54.707 |
| CHEMBL2105399 | SULFAMOXOLE | Sulfamoxole |  | Antibacterial |  | -54.422 |
| CHEMBL2105424 | ROMIFIDINE | Romifidine |  |  |  | -53.802 |
| CHEMBL2110857 | LAVOLTIDINE | AH-23844 FREE BASE | 2 | Anti-Ulcerative (histamine H2-receptor blocker) |  | -53.021 |
| CHEMBL157337 | RAMIFENAZONE | Ramifenazone |  |  | Adrenergic receptor beta | -52.573 |
| CHEMBL2107408 | GLYBUZOLE | AN 1324 |  |  |  | -52.114 |
| CHEMBL35057 | TIOSPIRONE | BMY-13859 |  | Antipsychotic | Dopamine D2 receptor | -50.376 |
| CHEMBL4297595 | LISAVANBULIN | Bal101553 | 1 |  |  | -79.752 |
| CHEMBL3545369 | EPACADOSTAT | Epacadostat | 3 |  | Indoleamine 2,3-dioxygenase | -74.617 |
| CHEMBL3544964 | RAVOXERTINIB | GDC0994 | 1 |  | MAP kinase ERK2 | -72.195 |
| CHEMBL3678958 | SKLB1028 | Sklb1028 | 3 |  | Vascular endothelial growth factor receptor 2 | -71.307 |
| CHEMBL114586 | SEPIMOSTAT | Sepimostat |  |  |  | -69.987 |
| CHEMBL1950289 | TANZISERTIB | CC-930 | 2 |  |  | -69.192 |
| CHEMBL2105110 | LAMTIDINE | AH 22216 |  |  |  | -68.838 |
| CHEMBL453 | **SULFISOXAZOLE** | Entusil | Approved | Antibacterial | Endothelin receptor ET-A | -65.234 |
| CHEMBL2104908 | SULFAZAMET | Sulfapyrazole |  | Antibacterial |  | -64.931 |
| CHEMBL2105508 | SULFATROZOLE | Sulfatrozole |  |  |  | -64.900 |
| CHEMBL2106717 | BUTADIAZAMIDE | Butadiazamide |  |  |  | -64.863 |
| CHEMBL2105528 | BISFENAZONE | Bisfenazone |  |  | Carboxylesterase | -64.020 |
| CHEMBL1108 | DROPERIDOL | Dridol | Approved | Antipsychotic | Serum albumin | -64.020 |
| CHEMBL1484857 | SUCCINYLSULFATHIAZOLE | Succinylsulfathiazole |  |  | Prelamin-A/C | -63.267 |
| CHEMBL3128043 | PF-03758309 | Pf-03758309 | 1 |  |  | -62.667 |
| CHEMBL2106134 | DALBRAMINOL | Dalbraminol |  |  |  | -61.849 |
| CHEMBL1355299 | SULFAETHIDOLE | Sulfaethidole |  |  | Putative fructose-1,6-bisphosphate aldolase | -60.013 |
| CHEMBL94087 | GLYBUTHIAZOL | Glybuthiazol |  |  | NON-PROTEIN TARGET | -59.442 |
| CHEMBL459505 | TALAROZOLE | R115866 | 2 |  | Cytochrome P450 26B1 | -58.339 |
| CHEMBL844 | BRIMONIDINE | AGN-190342 FREE BASE | Approved | Adrenergic (ophthalmic) | Oryctolagus cuniculus | -57.543 |
| CHEMBL2106654 | ETISAZOLE | BAY VA 9387 |  |  |  | -57.030 |
| CHEMBL1079 | TIZANIDINE | Tizanidine | Approved | Antispasmodic |  | -56.203 |
| CHEMBL3545378 | MK-6592 | Mk-6592 | 1 |  | Serine/threonine-protein kinase Aurora-B | -56.160 |
| CHEMBL2107088 | SULFASOMIZOLE | Sulfasomizole |  | Antibacterial |  | -54.348 |
| CHEMBL2103842 | VARLITINIB | AR-00334543 | 2 |  | Receptor protein-tyrosine kinase erbB-2 | -53.970 |
| CHEMBL1509115 | TETRYDAMINE | POLI 67 |  | Analgesic; Anti-Inflammatory |  | -53.035 |
| CHEMBL2008915 | GUAMECYCLINE | Guamecycline |  |  |  | -52.630 |
| CHEMBL1742413 | PIBUTIDINE | Pibutidine |  |  |  | -52.630 |
| CHEMBL2105738 | GALETERONE | Galeterone | 3 |  | LNCaP | -52.120 |
| CHEMBL26630 | SULFATROXAZOLE | Sulfatroxazole |  |  | Endothelin receptor ET-A | -51.967 |
| CHEMBL558752 | RAF-265 | CHIR-265 | 2 |  | Tyrosine-protein kinase receptor FLT3 | -51.796 |
| CHEMBL4582651 | PRALSETINIB | BLU123244 | Approved |  | Replicase polyprotein 1ab | -51.729 |
| CHEMBL12552 | BIMAKALIM | Bimakalim |  |  | Cavia porcellus | -51.276 |
| CHEMBL482968 | ENMD-2076 | Enmd 2076 | 2 |  | Serine/threonine-protein kinase Aurora-A | -78.088 |
| CHEMBL3545215 | BMS-911543 | Bms-911543 | 2 |  | Phosphodiesterase 4 | -69.917 |
| CHEMBL3422109 | AVANBULIN | Avanbulin |  |  |  | -67.283 |
| CHEMBL2178575 | GSK-2239633 | GSK2239633 | 1 |  | Canis familiaris | -67.128 |
| CHEMBL437 | **SULFATHIAZOLE** | Sulfathiazole | Approved | Antibacterial |  | -65.665 |
| CHEMBL1614710 | OSI-930 | Osi 930 | 1 |  | Stem cell growth factor receptor | -64.166 |
| CHEMBL4206033 | BAY1436032 | Bay1436032 | 1 |  | Isocitrate dehydrogenase [NADP] cytoplasmic | -62.532 |
| CHEMBL3989970 | MAVELERTINIB | Mavelertinib | 2 |  | Epidermal growth factor receptor erbB1 | -62.229 |
| CHEMBL296419 | ASTEMIZOLE | Astemizole | Approved | Antihistaminic; Anti-Allergic |  | -60.256 |
| CHEMBL383581 | EDONENTAN | BMS-207940-02 |  |  | Endothelin receptor ET-A | -58.930 |
| CHEMBL3545085 | XL-228 | XL228 | 1 |  | Insulin-like growth factor I receptor | -57.870 |
| CHEMBL4650285 | LY3214996 | [14c]-ly3214996 | 2 |  |  | -57.252 |
| CHEMBL51483 | GOSSYPOL | BL-193 | 3 |  | Plasmodium falciparum | -54.231 |
| CHEMBL2104845 | ONTAZOLAST | BIRM-270 |  | Anti-Asthmatic (leukotriene antagonist) |  | -54.079 |
| CHEMBL3809489 | BEMCENTINIB | Bemcentinib | 2 |  | MDA-MB-231 | -51.909 |
| CHEMBL25336 | **BISANTRENE** | Bisantrene | 3 |  | RPMI-8226 | -50.357 |
| CHEMBL575448 | BMS-754807 | Bms-754807 | 2 |  | Insulin-like growth factor I receptor | -71.693 |
| CHEMBL282724 | SITAXENTAN | IPI-1040 | Approved |  |  | -68.245 |
| CHEMBL3769414 | LENRISPODUN | IC200214 | 1 |  |  | -66.392 |
| CHEMBL4577523 | AZD4205 | Azd4205 | 2 |  | Tyrosine-protein kinase JAK1 | -62.662 |
| CHEMBL1956820 | GS-9256 | Gs-9256 | 2 |  | Hepatitis C virus | -59.114 |
| CHEMBL3544911 | PREXASERTIB | LY2606368 | 2 |  | SARS-CoV-2 | -53.630 |
| CHEMBL1191 | **SULFAMETHIZOLE** | Methazol | Approved | Antibacterial |  | -53.525 |
| CHEMBL4594428 | ASP-5878 | Asp5878 | 1 |  |  | -52.899 |

**Supplementary Table S2.** Cell survival and IFN-alpha secretion of *OASL*-transfected THP-1 cells. Cell culture was performed in the presence of the compounds with 6 different concentrations (0.1, 1, 10, 100, 250, 1000 μM). The concentrations of induction of 50% cell death (Cytotoxic IC50) and efficacy concentration of 50% inhibition of IFN-alpha secretion (EC50) were estimated by exponential regression. The therapeutic index (TI) was defined as the ratio of cytotoxic IC50 to EC50.

| Compound | Estimated cytotoxic IC50 | Estimated EC50 | TI |
| --- | --- | --- | --- |
| Phthalylsulfathiazole | 600(μM) | 90 (μM) | 6.7 |
| Bisantrene | 680 (nM) | 190 (nM) | 3.6 |
| Sulfisoxazole | 550 (μM) | 190 (μM) | 2.9 |
| Talarozole | 2.1 (nM) | 0.8 (nM) | 2.7 |
| Ganaplacide hydrochloride | 7.7 (nM) | 0.6 (nM) | 12.8 |
| Sulfathiazole sodium | 170 (μM) | 130 (μM) | 1.3 |
| Sulfamethizole | >1000 (μM) | 630 (μM) |  |
